# Treatment of Temporary Teeth

**Published:** 1889-01

**Authors:** H. Cowie


### COMMUNICATIONS.

### Treatment of Temporary Teeth.

BY DR. H. COWIE.

[Read before the Detroit Dental Society.]

Our worthy President in assigning as the subject for discussion for this meeting, "Treatment of Deciduous Teeth" must surely have had in mind the very limited knowledge we possess on this subject, and the very uncertain success that attends our efforts in doing any thing for many of the children that are brought to us for treatment. Probably nothing connected with our profession is less understood or appreciated by the general public, and indeed by very many of those who are in the profession than the Treatment of the Temporary Teeth, the practice of too many being to follow out the ideas held by those who bring the children, viz.: extract them to relieve either the pain the child may be suffering from, or to remedy irregularity of some incoming tooth. We all know how hard it is to operate for children and how difficult it is to do for them what we could wish, for we most generally are called on only when the child is suffering from toothache, for parents, even those who do give attention to their own teeth, and to those of their children whom they may deem of a proper age to come under the care of the dentist neglect, through ignorance, the temporary teeth and only call on us when compelled by failure on their part to alleviate or stop the pain the child may be suffering from the defective tooth or teeth. Then they are in many cases only anxious that we should remove the tooth or teeth on the plea that they are only temporary and of no possible account any way, not realizing the fact that if they are extracted the child will not have any thing to masticate with, or have at least a more defective apparatus with which to prepare their food properly than they should have, and can have, if the defects are made good. Then too we must bear in mind the effect the premature extraction may, or rather will surely have on the contraction of the jaws and the consequent irregularity of the permanent teeth. Of course it is our duty to use reasonable effort to instruct and convince the parents of the value of the temporary teeth and impress upon them the importance of their retention until the proper time for the eruption of the second, or permanent teeth.

I will not attempt to say any thing of the number of the deciduous teeth, their time of eruption, or do any thing more than to give so far as possible the method I follow in the treatment of these teeth.

My first effort in this direction is, if possible, to get the confidence of the little ones and do away with the idea that it is my only province to cause them pain; then having accomplished this, select some easy cavity, fill it, and dismiss them with a very short sitting, for they are easily hurt and do not stand long fatigue.

They will usually come for the second visit much more readily than at first and I can then do more even though the operation may be much more tedious and painful. Have found that Calk's Diamond Cement is just the thing to use in crown cavities as it sets quickly and will harden under water if kept dry for one minute. Have not found it so reliable in proximal cavities as it seems to fail by disintegrating at the margin of the cavity, near the gum tissue. If, however, the cavity does not go near the gum I use it in preference to amalgam, as in my hands amalgam does not seem to do so well as the cement, in large proximal cavities extending to both teeth. I use the common base plate gutta-percha and fill the two cavities together with one large piece making no effort to separate the teeth. The effect of this mode of filling with this material is sometimes to cause the teeth so treated to separate but this does not seem any objection in most cases, as it only enlarges the space for the incoming tooth, which is in most cases rather an advantage.

If the nerve is found exposed I usually apply creosote for a time to allay the pain, then use a very minute portion of arsenic on the second visit leaving it in only twelve hours, then take out the pulp not with a broach, but with a spoon-shaped excavator, then drill a hole just at the line of the gum to act as a vent for the tooth, place a piece of asbestos paper on the floor of the cavity saturated with creosote, then fill with the phosphate or amalgam. I do not claim any originality in thus operating, as in so doing I have only followed the course that has been advocated by others, and am merely giving it as the method I have been using.

There is one thing I do for which I expect to be perhaps sharply criticised, that is for the method I pursue when in cases where the permanent incisors are in place but in a crowded condition, and that is to remove the second temporary molar on each side. I speak of the upper jaw only as no cases have presented themselves that I can recall where it was deemed necessary in the lower maxillary.

The second temporary molar as you know is very much larger than the second bicuspid, which takes its place and the removal of this molar gives room, more particularly for the canine which is so very apt to be out of line for want of room, by allowing the first bicuspid to be forced back still giving room for the second bicuspid to take its proper position or if it does not I do not hesitate to extract them both thereby leaving the mouth in better form than if in a crowded irregular condition.

The regulation of teeth when out of position is a very painful tedious operation and one that if it can be avoided by such a simple thing as by the loss at the right time of two or even more teeth ought in justice to our patients to be done.

---

Menthol used as a stimulant, alterative and analgesic. Best applied locally in combination with oleum lanae. It is of value in phthisic in one and one-third grain doses internally.

